# Real‐world treatment patterns and clinical outcomes in patients with stage III NSCLC in Korea: The KINDLE study

**DOI:** 10.1002/cam4.7174

**Published:** 2024-04-15

**Authors:** Jiyun Lee, Hee Kyung Ahn, Sang‐We Kim, Ji‐Youn Han, Sung Sook Lee, Hyung Soon Park, Hyun Woo Lee, Joo‐Hang Kim, Eunhan Cho, Reto Huggenberger, Byoung Chul Cho

**Affiliations:** ^1^ Lung Cancer Center, Yonsei Cancer Center Yonsei University College of Medicine Seoul Korea; ^2^ Department of Medical Oncology Gachon University Gil Medical Center Incheon Korea; ^3^ Department of Oncology, Asan Medical Center University of Ulsan College of Medicine Seoul Korea; ^4^ Center for Lung Cancer, National Cancer Center Research Institute and Hospital Goyang Korea; ^5^ Department of Hematology‐Oncology Inje University Haeundae Paik Hospital Busan Korea; ^6^ Division of Medical Oncology, Department of Internal Medicine, St. Vincent's Hospital The Catholic University of Korea Suwon Korea; ^7^ Department of Hematology‐Oncology Ajou University School of Medicine Suwon Korea; ^8^ CHA Bundang Medical Center CHA University Seongnam Korea; ^9^ AstraZeneca Seoul Korea; ^10^ AstraZeneca Baar Switzerland

**Keywords:** chemoradiation, palliative therapy, stage III NSCLC, surgery

## Abstract

**Objective:**

KINDLE‐Korea is part of a real‐world KINDLE study that aimed to characterize the treatment patterns and clinical outcomes of patients with stage III non‐small cell lung cancer (NSCLC).

**Materials and Methods:**

The KINDLE was an international real‐world study that explores patient and disease characteristics, treatment patterns, and survival outcomes. The KINDLE‐Korea included stage III NSCLC patients diagnosed between January 2013 and December 2017.

**Results:**

A total of 461 patients were enrolled. The median age was 66 years (range: 24–87). Most patients were men (75.7%) with a history of smoking (74.0%), stage IIIA NSCLC (69.2%), and unresectable disease (52.9%). A total of 24.3% had activating EGFR mutation and 62.2% were positive for PDL1 expression. Broadly categorized, 44.6% of the patients received chemoradiation (CRT)‐based therapy, 35.1% underwent surgery, and 20.3% received palliative therapies as initial treatment. The most commonly adopted approaches for patients with stage IIIA and IIIB disease were surgery and CRT, respectively. The median PFS was 15.2 months and OS was 66.7 months. Age >65 years, adenocarcinoma histology, and surgery as the initial treatment were significantly associated with longer OS.

**Conclusion:**

This study revealed the heterogeneity of treatment patterns and survival outcomes in patients with stage III NSCLC before durvalumab consolidation came into clinical practice. There is an unmet need for patients who are not eligible for surgery as an initial therapy. Novel therapeutic approaches are highly warranted to improve clinical outcomes.

## 1 INTRODUCTION

Lung cancer is the most common cancer in Korea, with 42,323 new cases, and is the leading cause of cancer‐related deaths, with 31,945 deaths in 2020.^1^ In Korea, the incidence and deaths related to lung cancer are predominant in males and the elderly population with an age above 65 years.^2^

Non‐small cell lung cancer (NSCLC) accounts for approximately 85% of all lung cancer cases.^3^ Of these, 22.9% were diagnosed with stage III disease in Korea,^4^ a proportion similar to that of patients worldwide.^5^, ^6^ Stage III NSCLC represents a highly heterogeneous disease with diverse tumor and nodal status, therapeutic options, and prognoses.^7^ It was subdivided into stages IIIA and IIIB in the seventh edition of the American Joint Committee on Cancer (AJCC) classification system, until it was recategorized as stages IIIA, IIIB, and IIIC in the eighth edition, mainly according to the nodal involvement status.^8^, ^9^

The mortality rate of all‐stage lung cancer decreased from 22.5% (1999) to 15.6% (2019) over 20‐year period in Korea.^10^ Despite such improvement, the survival outcome for stage III NSCLC remains poor, with 5‐year relative survival rates of 33% and 5.2% for stages IIIA and IIIB, respectively, according to the seventh edition of the AJCC classification system.^11^ In another study evaluating long‐term survival in stage III NSCLC, patients who underwent complete resection had an overall survival (OS) rate of 50.9% at 5 years and 37.7% at 10 years compared with 15.5% and 2.8% for patients without complete resection, respectively.^12^

Traditionally, surgery with or without neoadjuvant concurrent chemoradiation (cCRT) has been the most commonly adopted therapeutic approach in Korea (49.6%), followed by definitive cCRT, palliative chemotherapy, and radiotherapy alone.^13^ The treatment patterns for stage III NSCLC varies widely, despite the recommended guidelines, attributable to the differences in tumor and patient status, treating physicians' inclinations, and availability of medical infrastructure.^4^, ^13^, ^14^, ^15^, ^16^

The treatment paradigm has shifted dramatically for stage III, locally advanced, unresectable NSCLC patients with the recent advent of durvalumab consolidation after definitive cCRT, demonstrating a significant benefit in progression‐free survival (PFS) and OS.^17^ Nonetheless, data on the treatment patterns and clinical outcomes in Korean patients with stage III NSCLC remain limited.

KINDLE was a real‐world, multinational study conducted to determine the treatment patterns and clinical outcomes of patients with stage III NSCLC before durvalumab consolidation was introduced into clinical practice.^18^ Here, we report the results of “KINDLE‐Korea,” a subgroup analyses on patients from Korea.

## 2 MATERIALS AND METHODS

### 2.1 Study design

The KINDLE‐Korea study was conducted across eight centers in South Korea as a subset of the global (non‐United States and non‐European Union), noninterventional KINDLE study.^18^ The independent ethics committees/institutional review boards of all participating centers approved the study protocol (NCT03725475). This study was conducted under the Helsinki Declaration, International Council for Harmonisation (ICH), Good Clinical Practices (GCP), Good Pharmacoepidemiology Practices (GPP), and relevant noninterventional and/or observational studies legislation.

### 2.2 Study population

This study included patients aged 18 years or older who were diagnosed with de novo locally advanced stage III NSCLC (according to the AJCC classification system, seventh edition) between January 2013 and December 2017. Patients were excluded if they had an initial diagnosis of stage I or II NSCLC that progressed to stage III disease, had concomitant cancer diagnosed within 5 years of the index date (except for nonmetastatic nonmelanoma skin cancers, or in situ or benign neoplasms), or had a follow‐up duration of <9 months since the initial diagnosis.

### 2.3 Data collection and study outcomes

Details on data collection and study outcomes have been reported previously.^18^ PFS was defined as the time from treatment initiation to documented disease progression or death due to any cause, whichever occurred first. OS was defined as the time from initial diagnosis or treatment initiation until death due to any cause.

### 2.4 Statistical analyses

Descriptive statistics were used to summarize the patient demographics, disease characteristics, and treatment patterns. Categorical variables were presented as percentages, while continuous variables as medians, minimums, and maximums. Median survival estimates (PFS and OS) were determined using the Kaplan–Meier method and compared using the log‐rank test. Median survival estimates were reported along with the two‐sided 95% confidence interval (CI). Univariate and multivariate models using relevant characteristics associated with PFS and OS were based on Cox proportional hazard regression analyses. All analyses were performed using SAS version 9.4. A *p*‐value ≤0.05 was considered statistically significant.

## 3 RESULTS

### 3.1 Patient characteristics

A total of 461 patients were enrolled, with a median follow‐up duration of 772.0 days (range: 4–2280 days). The baseline characteristics are summarized in Table 1. The median age of the patients was 66 years (range: 24–87). The majority were men (75.7%), ex‐ or current smokers (74.0%), and had an Eastern Cooperative Oncology Group (ECOG) status of 0 or 1 (92.2%). Most patients had stage IIIA (69.2%), unresectable disease (52.9%) with adenocarcinoma histology (48.8%).

**TABLE 1:** Baseline characteristics.

Parameters, *n* (%)	Number of patients (*N* = 461)
Age (years), median (range)	66.0 (24.0–87.0)
Gender, *n* (%)	
Male	349 (75.7)
Female	112 (24.3)
Smoking history^a^, *n* (%)	
Current smoker	147 (31.9)
Ex‐smoker	194 (42.1)
Never smoker	110 (23.9)
AJCC stage^b^, *n* (%)	
IIIA	312 (69.2)
IIIB	139 (30.8)
Resectability, *n* (%)	
Resectable	193 (41.9)
Unresectable	244 (52.9)
Histologic subtype, *n* (%)	
Adenocarcinoma	225 (48.8)
Squamous cell carcinoma	193 (41.9)
Large cell carcinoma	7 (1.5)
Other	22 (4.8)
ECOG performance status, *n* (%)	
0	99 (37.1)
1	147 (55.1)
≥2	21 (7.8)
T stage, *n* (%)	
T1	
T1a	13 (2.8)
T1b	38 (8.3)
T1c	1 (0.2)
T2	
T2a	135 (29.3)
T2b	46 (10.0)
T3	152 (33.0)
T4	73 (15.9)
N stage, *n* (%)	
N0	25 (5.4)
N1	45 (9.8)
N2	274 (59.6)
N3	116 (25.2)
EGFR mutational status, *n* (%)	
Tested	300 (65.1)
Mutant	73 (24.3)
Wildtype	220 (73.3)
Not tested	161 (34.9)
PD‐L1 expression status^c^, *n* (%)	
Tested	148 (32.1)
Positive	92 (62.2)
Negative	56 (37.8)
Not tested	313 (67.9)

*Note*: Unknown and missing data are not included.

Abbreviations: AJCC, American Joint Committee on Cancer; ECOG, Eastern Cooperative Oncology Group; EGFR, epidermal growth factor receptor; PD‐L1, programmed cell death‐ligand 1.

^a^ Current smoker was defined as an active smoker; ex‐smoker was defined as having smoked regularly but stopped ≥365 days ago; and never smoker was defined as never smoked regularly.

^b^ Stage according to AJCC Seventh edition.

^c^ PD‐L1 expression was tested using the Dako 22C3, Ventana SP263, or Ventana SP142 assays.

Overall, 41.9% of the patients had resectable disease. The majority (59.1%) of stage IIIA patients had resectable disease, whereas most patients with stage IIIB disease (90.1%) had unresectable disease (Table S1).

Of the 300 patients whose epidermal growth factor receptor (EGFR) mutational status was available, 24.3% had EGFR‐mutant tumor (Table 1). Patients with stage IIIA disease had a higher incidence of EGFR‐mutant tumors (28.4%) than those with stage IIIB disease (19.1%; Table S1). In terms of resectability, patients with resectable disease had a higher incidence of EGFR‐mutant tumor (33.1%) than those with unresectable disease (18.5%). While EGFR mutations were found more commonly in patients with resectable disease than in those with unresectable disease (33.9 vs. 18.2%) in stage IIIA group, the incidence was similar in stage IIIB group, regardless of resectability (22.2 vs. 18.8%, Table S1). The testing for PD‐L1 (programmed cell death ligand 1) expression was more commonly performed in patients with unresectable disease (38.1%) compared to those with resectable disease (26.1%). Overall, 62.2% (92 out of 148 tested) had a positive PD‐L1 expression (Table 1). A summary of the antibodies used for PD‐L1 testing is shown in Table S2.

### 3.2 Real‐world treatment patterns

**FIGURE 1:**
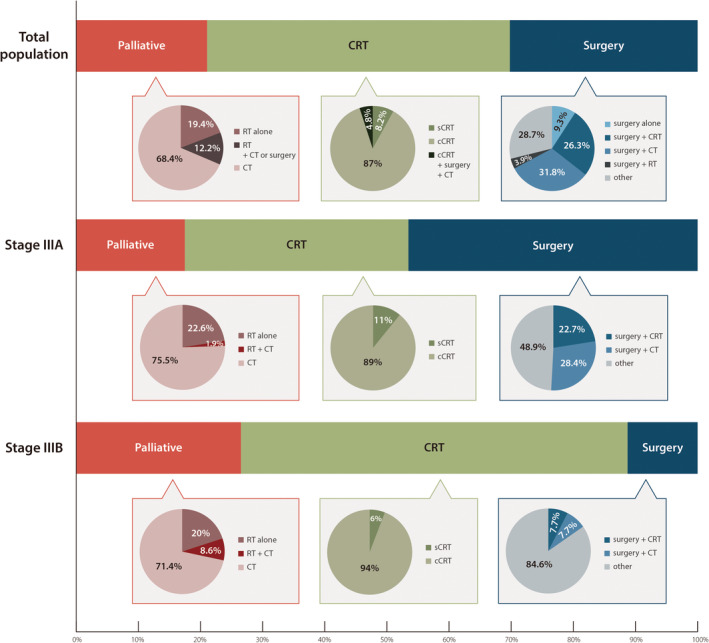
Treatment patterns for Korean patients with stage III NSCLC before the immunotherapy era. cCRT, concurrent chemoradiotherapy; CRT, chemoradiotherapy; CT, chemotherapy; NSCLC, non‐small cell lung cancer; RT, radiotherapy; sCRT, sequential chemoradiotherapy.

Of the 461 patients included in this study, 108 (23.4%) were approached using multidisciplinary team (MDT) discussion. Broadly categorized, 44.6% of the patients received chemoradiation (CRT)‐based, 35.1% received surgery‐based, and 20.3% received palliative therapies (including targeted, cytotoxic, and immunotherapeutic agents or palliative radiation) as initial treatment (Figure 1, Table S3). For patients with stage IIIA disease, the most commonly adopted therapeutic approach was surgery (46.5%), followed by CRT (36.0%) and palliative therapies (17.5%). For those with stage IIIB disease, CRT (63.6%) was the most commonly adopted treatment, followed by palliative therapy (26.5%) and surgery (9.8%, Figure 1, Table S3).

Over the study period, 283 (61.4%) patients experienced recurrent/relapsed disease after the initial treatment, including 101 of 193 (53.7%) patients with resectable disease and 182 of 244 (76.8%) patients with unresectable disease. Among them, 208 (73.5%) received second‐line therapy (Table S3). Understandably, most patients were treated with a palliative aim regardless of the initial stage at diagnosis, whereas a minority were treated with CRT‐based (18.8% and 14.1% as second‐ and third‐line therapies, respectively) or surgery‐based approaches (6.3% and 9.0% as second‐ and third‐line therapies, respectively). Detailed information is provided in Tables S4 and S5.

### 3.3 Survival outcomes

**FIGURE 2:**
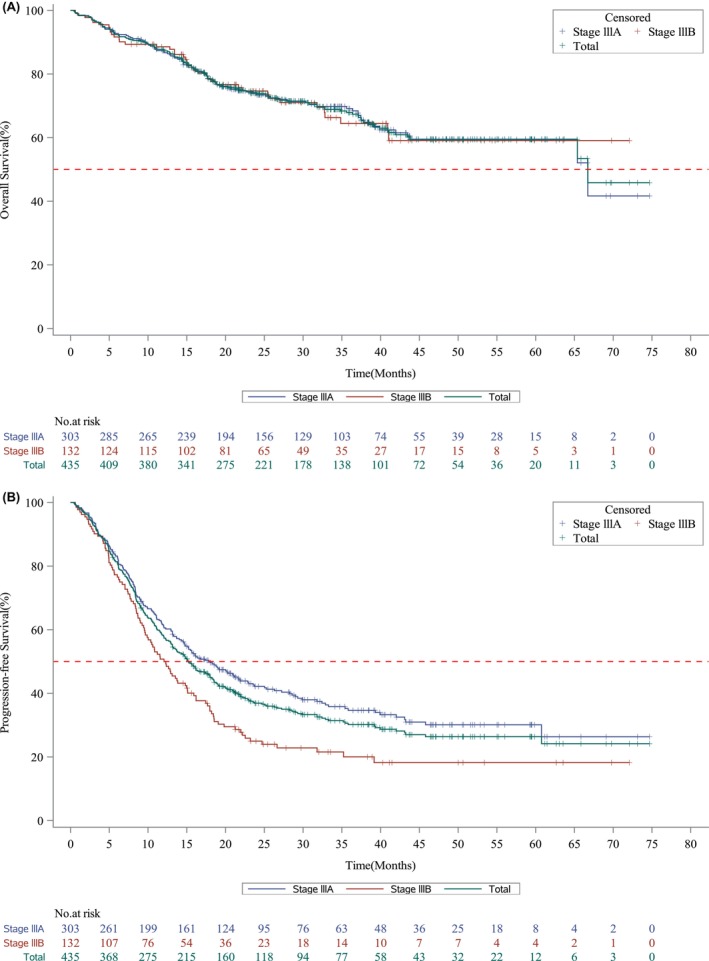
Kaplan–Meier survival curves for (A) overall and (B) progression‐free survival according to stage.

Approximately two thirds (68.1%) of patients were alive at the time of data collection. The median OS was 66.7 months (95% CI: 65.4–not calculable [NC]) for the total population (Figure 2A). The 5‐year OS rates for patients with stages IIIA and IIIB were 59.5% (95% CI: 57.0–61.9) and 59.1% (95% CI: 55.0–62.9), respectively. In resectable and unresectable disease, the 5‐year survival rates were 66.9% (95% CI: 63.8–69.7) and 53.8% (95% CI: 50.8–56.8), respectively. However, patients initially treated with the CRT‐based approach had the lowest 5‐year OS rate compared to those treated with surgery and palliative therapy: 39.2% (95% CI: 29.3–48.9) and 53.6% (95% CI: 49.9–57.1) in those receiving CRT‐based treatment with resectable and unresectable disease, respectively (Table 2).

**TABLE 2:** Overall survival according to stage, resectability, and initial treatment approach.

Overall survival	Median, months (95% CI)	OS rate, % (95% CI)		
		1‐year OS	3‐year OS	5‐year OS
Total population	66.7 (65.4 – NC)	87.7 (86.6–88.8)	67.9 (66.2–69.5)	59.4 (57.3–61.4)
Stage^a^				
IIIA	66.7 (65.4 – NC)	87.4 (86.0–88.6)	69.1 (67.1–71.0)	59.5 (57.0–61.9)
IIIB	NC (41.0 – NC)	88.6 (86.5–90.3)	64.4 (60.9–67.7)	59.1 (55.0–62.9)
Resectable^a^				
Overall	66.7 (65.4 – NC)	90.4 (88.8–91.7)	75.6 (73.3–77.8)	66.9 (63.8–69.7)
Surgery‐based therapy	35.8 (22.9 – NC)	89.9 (88.1–91.5)	79.5 (77.0–81.8)	70.3 (66.9–0.73.4)
CRT‐based therapy	10.7 (6.2–19.1)	92.3 (85.5–96.0)	39.2 (29.3–48.9)	39.2 (29.3–48.9)
Palliative therapy	19.2 (9.3–29.0)	92.0 (87.4–95.0)	71.6 (64.9–77.2)	57.3 (46.7–66.5)
Unresectable^a^				
Overall	NC (37.52 – NC)	86.4 (84.8–0.878)	60.5 (57.9–63.1)	53.8 (50.8–56.8)
Surgery‐based therapy	14.8 (7.8 – NC)	100.0 (100.0–100.0)	66.7 (51.9–77.8)	66.7 (51.9–77.8)
CRT‐based therapy	12.1 (9.4–14.8)	86.6 (84.7–88.2)	62.2 (59.1–65.2)	53.6 (49.9–57.1)
Palliative therapy	10.8 (7.9–12.7)	84.5 (81.0–87.4)	55.7 (50.5–60.5)	55.7 (50.5–0.60.5)

Abbreviations: CI, confidence interval; CRT, chemoradiotherapy; NC, not calculable; OS, overall survival.

^a^ Stage and resectability status data captured at the index date were used to determine the subgroups for subgroup analysis.

The median PFS from the time of initial treatment was 15.2 months (95% CI: 13.1–18.0) for the total population; 18.0 (95% CI: 14.7–21.7) and 12.2 (95% CI: 9.7–15.0) months for patients with stages IIIA and IIIB disease, respectively (Figure 2B). As summarized in Table 3, the difference in PFS duration increased according to resectability compared to stages, with median PFS of 26.3 (95% CI: 20.2–40.0) and 11.1 (95% CI: 9.4–13.1) months for those with resectable and unresectable disease, respectively. The initial surgery‐based approach demonstrated the longest PFS compared to the other approaches, although it differed significantly according to stage and resectability. In contrast, the CRT‐based approach demonstrated a consistent duration of PFS ranging between 10.5 and 12.9 regardless of stages or resectability.

**TABLE 3:** Progression‐free survival according to the initial treatment approach.

First‐line treatment	Median PFS, months (95% CI)			
	Stage IIIA (*n* = 303)	Stage IIIB (*n* = 132)	Resectable (*n* = 188)	Unresectable (*n* = 237)
Overall	18.0 (14.7–21.7)	12.2 (9.7–15.0)	26.3 (20.2–40.0)	11.1 (9.4–13.1)
Surgery‐based therapy	29.9 (21.1 – NC)	NC (18.2 – NC)	35.8 (22.9 – NC)	14.8 (7.8 – NC)
CRT‐based therapy	12.9 (8.9–17.8)	10.5 (8.7–13.8)	10.7 (6.2–19.1)	12.1 (9.4–14.8)
Palliative therapy	12.0 (8.4–16.1)	11.5 (4.9–14.8)	19.2 (9.3–29.0)	10.8 (7.9–12.7)

*Note*: Staging according to the American Joint Committee on Cancer Seventh Edition.

Abbreviations: CI, confidence interval; CRT, chemoradiotherapy; NC, not calculable; and PFS, progression‐free survival.

Univariate and multivariate survival analyses were performed using clinically relevant variables and initial treatment modalities (Table 4). In the multivariate analysis, age of more than 65 years, adenocarcinoma histology, and surgery as initial therapy were significantly associated with longer OS (all *p* < 0.05), while only surgery as initial treatment approach was significantly associated with a longer PFS (hazard ratio: 0.42, 95% CI: 0.28–0.63, *p* < 0.05).

**TABLE 4:** Univariate and multivariate analyses for overall and progression‐free survivals.

	Univariate analysis			Multivariate analyses	
	HR (95% CI)	*p*‐Value		HR (95% CI)	*p*‐Value
Overall survival					
Age >65 versus ≤65 years (222 vs. 222)	1.799 (1.280–2.527)	**0.0007**	Age >65 versus ≤65 (222 vs. 222)	1.538 (1.086–2.177)	**0.0153**
Male versus female (335 vs. 109)	1.911 (1.221–2.992)	**0.0046**	Male versus Female (335 vs. 109)	1.439 (0.874–2.369)	0.1522
Adenocarcinoma histology versus others (219 vs. 225)	0.525 (0.372–0.740)	**0.0002**	Adenocarcinoma histology versus Others (219 vs. 225)	0.615 (0.419–0.902)	**0.0129**
Surgery in first line yes versus no (156 vs. 288)	0.541 (0.370–0.790)	**0.0015**	Surgery in first line yes versus no (156 vs. 288)	0.526 (0.281–0.987)	**0.0453**
CRT in first line yes versus no (267 vs. 177)	0.960 (0.684–1.347)	0.8129	CRT in first line yes versus no (267 vs. 177)	0.659 (0.330–1.315)	0.2365
Palliative therapy in first line yes versus no (90 vs. 354)	1.343 (0.904–1.995)	0.1437	Palliative therapy in first line yes versus no (90 vs. 354)	0.867 (0.381–1.975)	0.7340
Progression‐free survival					
Age >65 versus ≤65 (222 vs. 222)	1.494 (1.188–1.878)	**0.0006**	Age >65 versus ≤65 (222 vs. 222)	1.242 (0.981–1.573)	0.0712
Male versus female (335 vs. 109)	1.187 (0.907–1.552)	0.2111	Male versus female (335 vs. 109)	1.072 (0.788–1.459)	0.6577
Adenocarcinoma histology versus Others (219 vs. 225)	0.838 (0.667–1.053)	0.1286	Adenocarcinoma histology versus Others (219 vs. 225)	0.931 (0.712–1.217)	0.6010
Surgery in first line yes versus no (156 vs. 288)	0.433 (0.334–0.563)	**<0.0001**	Surgery in first line yes versus no (156 vs. 288)	0.420 (0.281–0.627)	**<0.0001**
CRT in first line yes versus no (267 vs. 177)	1.039 (0.822–1.313)	0.7484	CRT in first line yes versus no (267 vs. 177)	0.786 (0.497–1.243)	0.3029
Palliative therapy in first line yes versus no (90 vs. 354)	1.563 (1.192–2.050)	**0.0012**	Palliative therapy in first line yes versus no (90 vs. 354)	0.909 (0.521–1.587)	0.7377

*Note*: Values in bold are statistically significant (*p* < 0.05).

Abbreviations: CI, confidence interval; CRT, chemoradiotherapy; and HR, hazard ratio.

For the 73 patients diagnosed with EGFR‐mutant NSCLC, the primary first‐line treatment choice was surgery‐based therapy (49.3%), followed by palliative therapy (29.0%) and CRT‐based therapy at (21.7%). In terms of PFS, surgery exhibited the longest median PFS of 43.2 months (95% CI: 21.72–60.75). Notably, palliative therapy demonstrated a prolonged median PFS of 20.1 months (95% CI: 11.99–23.72) compared to CRT‐based therapy, which had a median PFS of 6.2 months (95% CI: 3.29–15.24). While the sample sizes in these subsets are limited to reach definitive conclusions, it is noteworthy that the observed PFS trends did not align with OS outcomes. Surgery and palliative therapy demonstrated a median OS of 66.7 (95% CI: NC–NC) and 65.4 (95% CI 65.38–NC) months, respectively, whereas the median OS for CRT‐based therapy was not reached (Tables S6–S8).

## 4 DISCUSSION

This retrospective, real‐world study on patients with stage III NSCLC is one of the largest studies of its kind, including 461 Korean patients. Our data provide insights into the treatment approaches and their associated survival outcomes before the era of durvalumab.^19^ It provides a reference point for the clinical unmet needs before the introduction of durvalumab consolidation and the associated benefits that can come along with it.

A higher number of patients were first diagnosed through a cancer screening program in the Korean subset (18.9%) than in the global cohort (4.1%).^18^ This may be attributable to the implementation of the Korean Lung Cancer Screening Project as a nationwide program, which recommended the use of low‐dose computed tomography for high‐risk patients defined as aged 55–74 years with a smoking history of at least 30 pack‐years.^20^ Furthermore, cancer screening tests are commonly performed as part of employee health checkups. As such, the proportion of patients diagnosed with stage IIIA disease was higher in the Korean subset (69.2%) than in the global KINDLE cohort (55.9%), which could have led to a longer OS of 66.7 months compared to that of 34.9 months for the global KINDLE cohort.^18^

We found substantial diversity in treatment patterns, with at least 20 different approaches used as initial therapy. Approximately 80% of the patients received curative intent therapy, including surgery‐ or CRT‐based therapies. The most commonly adopted treatment modality was cCRT (34.5%). This is comparable to the results of the global KINDLE data, which reported that cCRT was the most common treatment modality (29.4%).^18^ Of note, the proportion of patients treated with surgery‐based therapy was higher in the Korean subset (31.5%) than in the global KINDLE cohort (21.4%).^18^ This might be due to the higher number of patients diagnosed with stage IIIA NSCLC in the Korean subset than in the global cohort.

Patient survival outcomes were affected by resectability and initial treatment modality. The number of patients with resectable disease treated with CRT‐based or palliative therapies implies that other factors, such as the patient's underlying comorbidities or preference, commonly affect the choice of the initial treatment modality. Multivariate analyses demonstrated that OS was significantly longer in patients who were initially treated with surgery than in those who were not. Consistent with our findings, a real‐world study in the Korean population also reported that patients in the surgical group had better survival rates than those in the nonsurgical group.^21^ The relatively poor survival outcomes of CRT‐based (with lack of effective consolidation treatment) or palliative therapies compared to surgery‐based therapy warrant a novel strategy of nonsurgical approaches to achieve better disease control.

The decision on the initial treatment of patients with stage III NSCLC became evermore complex since the establishment of post‐CRT durvalumab maintenance therapy as the standard practice. This mandated MDT discussions, which were officially introduced in Korea and encouraged by the National Health Insurance Service in August 2014, to optimize patient survival outcomes.^22^ One fourth (23.4%) of the cases were discussed at the MDT meeting, including 29.3% of the patients with stage IIIA disease and 17.0% of the patients with stage IIIB disease. The number of patients undergoing MDT‐led decision‐making is increasing and selecting an optimal treatment strategy is expected to improve patient outcomes.

The incidence of EGFR‐mutant NSCLC is more prevalent in the Asia‐Pacific region (36.8%–51.4%), including Korea, than in the Western regions (7%–22%).^14^, ^23^, ^24^ In our Korean cohort, 24.3% of patients with stage III NSCLC harbored EGFR mutations. Although cCRT followed by durvalumab maintenance is recommended, regardless of EGFR mutation status, for patients with stage III NSCLC, a recent exploratory post hoc subgroup analysis in a limited sample of 35 patients with EGFR mutations from the PACIFIC trial demonstrated similar PFS and OS between patients treated with durvalumab and placebo.^25^ In another retrospective analysis of 37 patients with unresectable stage III EGFR‐mutated NSCLC, PFS was not significantly different between patients who received durvalumab and those who received CRT alone (10.3 months vs. 6.9 months).^26^ In advanced NSCLC, a lack of efficacy of immune checkpoint inhibitors in patients with EGFR‐mutant NSCLC has been demonstrated in both tyrosine kinase inhibitor (TKI)‐naïve and ‐resistant settings.^27^, ^28^ Prospective data on the use of EGFR TKIs for patients with EGFR‐mutant stage III unresectable NSCLC are not yet available. The use of EGFR TKIs may be considered as palliative therapy in patients who have unresectable disease or are clinically unfit for surgery, if not administered as part of existing curative‐intent treatment regimens. Hence, it is critically important to address whether the identification of EGFR alteration(s) and subsequent treatment with EGFR‐TKIs as part of curative‐intent treatment strategies affect the treatment outcome in patients with locally advanced NSCLC. The ongoing LAURA trial (NCT03521154) is evaluating the efficacy of a third‐generation EGFR‐TKI, osimertinib, as maintenance treatment for patients with unresectable, EGFR‐mutant, stage III NSCLC who have not progressed following CRT treatment.^29^ Another ongoing trial (NCT04951635) is evaluating almonertinib, a third‐generation EGFR‐TKI, after CRT in patients with stage III unresectable NSCLC.^30^ Results are awaited for both trials.

An inherent limitation of our study lies in its retrospective nature. The results of the subgroup analyses should be interpreted with caution because of the small sample size. Further, the definition of resectability could not be specified because the decisions were made on the basis of individual patient by different physicians and institutions over an extended time period. Nonetheless, data from our KINDLE‐Korea cohort could serve as a reference to evaluate treatment outcomes in patients with stage III NSCLC after the establishment of durvalumab maintenance as a standard practice of care. The transition in the treatment paradigm for stage III NSCLC may be even more complex with the high incidence of EGFR‐mutant NSCLC in Korea.

## AUTHOR CONTRIBUTIONS

**Jiyun Lee:** Conceptualization (equal); data curation (lead); formal analysis (lead); investigation (lead); validation (lead); visualization (lead); writing – original draft (lead); writing – review and editing (lead). **Hee Kyung Ahn:** Conceptualization (equal); data curation (equal); formal analysis (equal); investigation (equal); supervision (equal); writing – review and editing (equal). **Sang‐We Kim:** Conceptualization (equal); formal analysis (equal); investigation (equal); methodology (equal); supervision (equal); validation (equal); writing – review and editing (equal). **Ji‐Youn Han:** Conceptualization (equal); data curation (equal); formal analysis (equal); funding acquisition (equal); investigation (equal); methodology (equal); supervision (equal); writing – review and editing (equal). **Sung Sook Lee:** Conceptualization (equal); data curation (equal); formal analysis (equal); investigation (equal); supervision (equal); writing – review and editing (equal). **Hyung Soon Park:** Conceptualization (equal); data curation (equal); formal analysis (equal); investigation (equal); supervision (equal); writing – review and editing (equal). **Hyun Woo Lee:** Conceptualization (equal); formal analysis (equal); investigation (equal); supervision (equal); writing – review and editing (equal). **Joo‐Hang Kim:** Conceptualization (equal); formal analysis (equal); investigation (equal); supervision (equal); writing – review and editing (equal). **Eunhan Cho:** Conceptualization (equal); funding acquisition (equal); project administration (equal); resources (equal); software (equal). **Reto huggenberger:** Conceptualization (equal); funding acquisition (equal); project administration (equal); resources (equal); software (equal). **Byoung Chul Cho:** Conceptualization (lead); data curation (lead); formal analysis (lead); funding acquisition (lead); investigation (lead); resources (lead); supervision (lead); writing – review and editing (lead).

## ETHICS STATEMENT

This study was conducted under the Helsinki Declaration, International Council for Harmonisation (ICH), Good Clinical Practices (GCP), Good Pharmacoepidemiology Practices (GPP), and relevant noninterventional and/or observational studies legislation. This study was approved by the Institutional Review Board of each participating institution with a waiver for informed consent.

## Supporting information


Table S1.

## Data Availability

The authors agree to make the data and materials supporting the results or analyses presented in this article available upon request.
