# Supplementary material for: Real‐world treatment patterns and clinical outcomes in patients with stage III NSCLC in Korea: The KINDLE study
Source: Cancer Med. 2024 Apr 15;13(7):e7174. doi: 10.1002/cam4.7174 (PMC11019151; doi:10.1002/cam4.7174)
Supplement: Supplementary file 1 — Table S1. [file CAM4-13-e7174-s001.docx]

**Supplementary Materials**

**Supplementary Table S1. EGFR mutation and PD-L1 expression status for patients with stage III NSCLC in Korea according to stage and resectability**

| **N = 427** | **Total**  **N = 427** | | **Stage IIIA**  **n = 296** | | **Stage IIIB**  **n = 131** | |
| --- | --- | --- | --- | --- | --- | --- |
| **Resectability** | **Resectable**  **(n = 188)** | **Unresectable**  **(n = 239)** | **Resectable**  **(n = 175)** | **Unresectable**  **(n = 121)** | **Resectable**  **(n = 13)** | **Unresectable**  **(n = 118)** |
| EGFR Testing, n (%) | 133 (70.7) | 146 (61.1) | 124 (70.9) | 66 (54.5) | 9 (69.2) | 80 (67.8) |
| Mutation | 44 (33.1) | 27 (18.5) | 42 (33.9) | 12 (18.2) | 2 (22.2) | 15 (18.8) |
| Wild type | 84 (63.2) | 117 (80.1) | 77 (62.1) | 53 (80.3) | 7 (77.8) | 64 (80.0) |
| PD-L1 Testing, n (%) | 49 (26.1) | 91 (38.1) | 45 (25.7) | 47 (38.8) | 4 (30.8) | 44 (37.3) |
| Positive | 32 (65.3) | 55 (60.4) | 29 (64.4) | 25 (53.2) | 3 (75.0) | 30 (68.2) |
| Negative | 17 (34.7) | 36 (39.6) | 16 (35.6) | 22 (46.8) | 1 (25.0) | 14 (31.8) |

EGFR, epidermal growth factor receptor; NSCLC, non-small cell lung cancer; PD-L1, programmed cell death‑ligand 1.

**Supplementary Table S2. Antibodies Used for PD-L1 Testing**

| **PD-L1 Antibody Used [n(%)]** | **Number of Patients (N=146)** |
| --- | --- |
| Dako22C3 | 22 (15.1%) |
| Dako22C3+Ventana SP263 | 14 (9.6%) |
| Dako22C3+Ventana SP263+Ventana SP142 | 3 (2.1%) |
| Dako 28-8 | 0 |
| Ventana SP263 | 46 (31.5%) |
| Ventana SP263+Ventana SP142 | 1 (0.7%) |
| Ventana SP263+Ventana SP142+Others | 1 (0.7%) |
| Ventana SP263+Others | 13 (8.9%) |
| Ventana SP142 | 14 (9.6%) |
| Others | 32 (21.9%) |
| Missing | 315 |

PD-L1, programmed cell death‑ligand 1

**Supplementary Table S3. First-line treatment patterns according to stage**

|  | **Overall**  **(N = 444)** | **Stage IIIA**  **(n = 296)** | **Stage IIIB**  **(n = 131)** |
| --- | --- | --- | --- |
| **Surgery** |  |  |  |
| Sx alone | 12 (2.7) | 0 | 0 |
| Sx+cCRT | 10 (2.3) | 0 | 0 |
| Sx+sCRT | 33 (7.4) | 32 (10.6) | 1 (0.8) |
| Sx+CT | 41 (9.2) | 40 (13.2) | 1 (0.8) |
| Sx+RT | 5 (1.1) | 0 | 0 |
| Other Sx | 37 (8.3) | 69 (22.8) | 11 (8.3) |
| **CRT** |  |  |  |
| cCRT+Sx+CT | 10 (2.3) | 0 | 0 |
| cCRT | 153 (34.5) | 86 (28.4) | 62 (47.0) |
| cCRT+CT | 14 (3.2) | 4 (1.3) | 10 (7.6) |
| Other cCRT | 14 (3.2) | 7 (2.3) | 7 (5.3) |
| sCRT | 16 (3.6) | 11 (3.6) | 5 (3.8) |
| Other sCRT | 1 (0.2) | 1 (0.3) | 0 |
| **Palliative** |  |  |  |
| RT+Sx | 8 (1.8) | 0 | 0 |
| CT | 51 (11.5) | 33 (10.9) | 16 (12.1) |
| CT+IO | 1 (0.2) | 1 (0.3) | 0 |
| RT | 19 (4.3) | 12 (4.0) | 7 (5.3) |
| RT+IO | 1 (0.2) | 1 (0.3) | 0 |
| RT+Targeted Therapy | 3 (0.7) | 0 | 3 (2.3) |
| Targeted Therapy | 15 (3.4) | 6 (2.0) | 9 (6.8) |

Note: stage according to American Joint Committee on Cancer staging 7^th^ edition.

cCRT, concurrent chemoradiotherapy; CT, chemotherapy; IO, immunotherapy; RT, radiotherapy; sCRT,  sequential chemoradiotherapy; Sx,  surgery.

**Supplementary Table S4. Treatment patterns in stage III NSCLC**

| **Treatment Modality** | **Initial Therapy, n (%)**  **(N = 444)** | **Recurrent/relapsed** | | |
| --- | --- | --- | --- | --- |
|  |  | **Second-Line, n (%)**  **(N = 208)** | **Third-Line, n (%)**  **(N = 78)** | |
| Sx alone | 12 (2.7) | 3 (1.4) | | 3 (3.8) |
| Sx+cCRT | 1 (2.3) | 0 | | 0 |
| Sx+sCRT | 33 (7.4) | 0 | | 0 |
| Sx+CT | 41 (9.2) | 0 | | 0 |
| Sx+RT | 5 (1.1) | 4 (1.9) | | 1 (1.3) |
| cCRT+Sx+CT | 10 (2.3) | 0 | | 0 |
| RT+Sx | 8 (1.8) | 2 (1.0) | | 0 |
| Other Sx | 37 (8.3) | 4 (1.9) | | 3 (3.8) |
| cCRT | 153 (34.5) | 18 (8.7) | | 2 (2.6) |
| cCRT+CT | 14 (3.2) | 2 (1.0) | | 2 (2.6) |
| Other cCRT | 14 (3.2) | 1 (0.5) | | 0 |
| sCRT | 16 (3.6) | 11 (5.3) | | 5 (6.4) |
| Other sCRT | 1 (0.2) | 7 (3.4) | | 2 (2.6) |
| CT | 51 (11.5) | 64 (30.8) | | 24 (30.8) |
| CT+IO | 1 (0.2) | 7 (3.4) | | 0 |
| RT | 19 (4.3) | 32 (15.4) | | 12 (15.4) |
| RT+IO | 1 (0.2) | 2 (1.0) | | 0 |
| RT+Targeted Therapy | 3 (0.7) | 5 (2.4) | | 2 (2.6) |
| Targeted Therapy | 15 (3.4) | 32 (15.4) | | 11 (14.1) |
| IO | 0 | 14 (6.7) | | 7 (9.0) |
| CT+Targeted Therapy | 0 | 0 | | 1 (1.3) |
| CT+Targeted Therapy+IO | 0 | 0 | | 1 (1.3) |
| RT+Targeted Therapy+IO | 0 | 0 | | 1 (1.3) |
| Targeted Therapy+IO | 0 | 0 | | 1 (1.3) |

Note: stage according to American Joint Committee on Cancer staging 7^th^ edition.

cCRT, concurrent chemoradiotherapy; CT, chemotherapy; IO, immunotherapy; RT, radiotherapy; sCRT,  sequential chemoradiotherapy; Sx:  surgery.

**Supplementary Table S5. Treatment patterns per resectability**

| **Treatment Modality** | **Resectable (N=193)** | **Unresectable (N=244)** |
| --- | --- | --- |
| **First-Line, n (%)** | | |
| Surgery+SCRT | 33 (17.6%) | 0 |
| Surgery+Chemotherapy | 40 (21.3%) | 1 (0.4%) |
| Other Surgery | 77 (41.0%) | 5 (2.1%) |
| CCRT | 5 (2.7%) | 138 (58.2%) |
| CCRT+Chemotherapy | 1 (0.5%) | 13 (5.5%) |
| Other CCRT | 2 (1.1%) | 12 (5.1%) |
| SCRT | 5 (2.7%) | 9 (3.8%) |
| Other SCRT | 0 | 0 |
| Chemotherapy | 22 (11.7%) | 26 (11.0%) |
| Chemotherapy+Immunotherapy | 1 (0.5%) | 0 |
| Radiotherapy | 1 (0.5%) | 17 (7.2%) |
| Radiotherapy+Immunotherapy | 0 | 1 (0.4%) |
| Radiotherapy+Targeted Therapy | 0 | 3 (1.3%) |
| Targeted Therapy | 1 (0.5%) | 12 (5.1%) |
| Total | 188 | 237 |
| **Second Line, n (%)** | | |
| Other Surgery | 8 (11.3%) | 5 (4.0%) |
| CCRT | 6 (8.5%) | 11 (8.7%) |
| CCRT+Chemotherapy | 0 | 2 (1.6%) |
| Other CCRT | 1 (1.4%) | 0 |
| SCRT | 3 (4.2%) | 6 (4.8%) |
| Other SCRT | 2 (2.8%) | 5 (4.0%) |
| Chemotherapy | 15 (21.1%) | 42 (33.3%) |
| Chemotherapy+Immunotherapy | 1 (1.4%) | 6 (4.8%) |
| Radiotherapy | 14 (19.7%) | 18 (14.3%) |
| Radiotherapy+Immunotherapy | 1 (1.4%) | 1 (0.8%) |
| Radiotherapy+Targeted Therapy | 4 (5.6%) | 0 |
| Immunotherapy | 0 | 14 (11.1%) |
| Targeted Therapy | 16 (22.5%) | 16 (12.7%) |
| Total | 71 | 126 |
| **Third Line, n (%)** | | |
| Other Surgery | 4 (14.3%) | 3 (6.5%) |
| CCRT | 1 (3.6%) | 1 (2.2%) |
| CCRT+Chemotherapy | 2 (7.1%) | 0 |
| SCRT | 0 | 5 (10.9%) |
| Other SCRT | 0 | 2 (4.3%) |
| Chemotherapy | 9 (32.1%) | 13 (28.3%) |
| Chemotherapy+Targeted Therapy | 1 (3.6%) | 0 |
| Chemotherapy+Targeted Therapy+Immunotherapy | 1 (3.6%) | 0 |
| Radiotherapy | 4 (14.3%) | 7 (15.2%) |
| Radiotherapy+Targeted Therapy | 0 | 1 (2.2%) |
| Radiotherapy+Targeted Therapy+Immunotherapy | 1 (3.6%) | 0 |
| Immunotherapy | 1 (3.6%) | 6 (13.0%) |
| Targeted Therapy | 4 (14.3%) | 7 (15.2%) |
| Targeted Therapy+Immunotherapy | 0 | 1 (2.2%) |
| Total | 28 | 46 |

cCRT, concurrent chemoradiation; sCRT, sequential chemoradiation.

**Supplementary Table S6.** Summary of treatment patterns according to EGFR status

|  | **Mutation**  **(N=73)** | **No mutation**  **(N=220)** |
| --- | --- | --- |
| **First Line[n(%)]** | 69 | 212 |
| Surgery Based therapy | 34 (49.3%) | 76 (35.8%) |
| CRT Based therapy | 15 (21.7%) | 87 (41.0%) |
| Palliative therapy | 20 (29.0%) | 49 (23.1%) |
|  |  |  |
| **Second Line[n(%)]** | 34 | 111 |
| Surgery Based therapy | 3 (8.8%) | 7 (6.3%) |
| CRT Based therapy | 2 (5.9%) | 20 (18.0%) |
| Palliative therapy | 29 (85.3%) | 84 (75.7%) |
|  |  |  |
| **Third Line[n(%)]** | 15 | 39 |
| Surgery Based therapy | 1 (6.7%) | 4 (10.3%) |
| CRT Based therapy | 2 (13.3%) | 4 (10.3%) |
| Palliative therapy | 12 (80.0%) | 31 (79.5%) |

CRT, chemoradiation.

**Supplementary Table S7.** Summary of PFS with different treatment pattern according to EGFR status.

|  |  | **Patient number** | |  |  | **Median PFS months**  **(95%CI)** | |  |
| --- | --- | --- | --- | --- | --- | --- | --- | --- |
| **First line treatment** | **Mutation** | | **No mutation** | | **Mutation** | | **No mutation** | |
| Surgery Based therapy | 34 | | 76 | | 43.2 (21.72 – 60.75) | | 24.7 (16.07 – NC) | |
| CRT Based therapy | 15 | | 87 | | 6.2 (3.29 – 15.24) | | 10.5 (8.64 – 14.82) | |
| Palliative therapy | 20 | | 49 | | 20.1 (11.99 – 23.72) | | 10.8 (7.89 – 12.02) | |

CI, confidence interval; CRT, chemoradiation; NC, not calculable.

**Supplementary Table S8.** Summary of OS with different treatment pattern according to EGFR status.

|  |  | **Patient number** | |  |  | **Median OS months (95%CI)** | |  |
| --- | --- | --- | --- | --- | --- | --- | --- | --- |
| **First line treatment** | **Mutation** | | **No mutation** | | **Mutation** | | **No mutation** | |
| Surgery Based therapy | 34 | | 76 | | 66.7 (NC – NC) | | NC (39.89 – NC) | |
| CRT Based therapy | 15 | | 87 | | NC (26.61 – NC) | | NC (36.37 – NC) | |
| Palliative therapy | 20 | | 49 | | 65.4 (65.38 – NC) | | NC (21.65 – NC) | |

CI, confidence interval; CRT, chemoradiation; NC, not calculable.
